# Oral health knowledge, behavior, and care seeking among pregnant and recently-delivered women in rural Nepal: a qualitative study

**DOI:** 10.1186/s12903-018-0564-9

**Published:** 2018-06-01

**Authors:** A. J. Lubon, D. J. Erchick, S. K. Khatry, S. C. LeClerq, N. K. Agrawal, M. A. Reynolds, J. Katz, L. C. Mullany

**Affiliations:** 1 Department of International Health, Johns Hopkins Bloomberg School of Public Health, 615 N. Wolfe Street W5009, Baltimore, MD 21205 USA; 2 Nepal Nutrition Intervention Project – Sarlahi (NNIPS), Krishna Galli, Lalitpur, Kathmandu Nepal; 3 Department of Dentistry, Institute of Medicine, Tribhuhvan University, Kathmandu, Nepal; 4 Department of Periodontics, University of Maryland School of Dentistry, Baltimore, MD USA

**Keywords:** Nepal, Periodontal disease, Oral health, Oral health behaviors, Dental care seeking behavior, Pregnancy

## Abstract

**Background:**

Oral health behavior and attitudes of pregnant women in low-income countries are rarely examined, yet should be considered when designing preventative or therapeutic studies to reduce burden of oral diseases. We aimed to understand dental care-seeking behavior, as well as oral health knowledge and attitudes of oral health among pregnant women in rural Nepal.

**Methods:**

Semi-structured in-depth interviews (*n* = 16) and focus group discussions (3 groups, *n* = 23) were conducted among pregnant and recently-delivered women in Sarlahi, Nepal. Transcripts were translated from the local language to English then analyzed using a hybrid approach to thematic coding with Atlas.ti version 7.

**Results:**

Women felt confident describing the signs and symptoms of tooth decay and gum disease, but were not knowledgeable about where to receive care for tooth and/or gum pain and relied heavily on the knowledge of their community. Some women used a toothbrush and toothpaste at least once a day to clean their teeth, but many reported the traditional use of a branch of a local shrub or tree as their teeth cleaning instrument. Women suggested a willingness to consider using an oral rinse throughout pregnancy, perceiving that it might have a positive impact on infant health.

**Conclusions:**

Future studies should focus on providing adequate and sustainable resources for pregnant women in Nepal and other low income settings to engage in good oral health behaviors (possibly supported through community-based workers), to maintain dental hygiene, and to access qualified dentists as a means of improving their oral health.

**Trial registration:**

ClinicalTrials.gov Identifier: NCT01177111 (Nepal Oil Massage Study) and NCT02788786 (Pilot Trial).

## Background

In low resource settings, oral health services are frequently inadequate to meet the needs of the population [1]. Poor accessibility, low quality, and human resources gaps – all key challenges to improving health systems in such settings – are more acutely felt for oral health systems. The limited capacity and insufficient distribution of trained oral health providers leads to utilization patterns almost exclusively focused on pain management or emergency treatment responses, at the expense of prevention [1]. In addition to supply side approaches that call for substantive human, infrastructural, and financial resources, demand side approaches require understanding of community norms, attitudes, practices, and knowledge toward oral health hygiene behavior, preventative care, and care-seeking practices.

Nepal is one of many low- and middle-income countries where attention and resources applied to oral health have lagged behind other domains of public health. Most oral health studies conducted in Nepal have tended to focus on burden of disease among school-aged children [2–7] or descriptive characteristics of dental professionals [8–10]. One study used a more broadly focused cross-sectional population-representative survey to identify factors associated with improved practices. Researchers found that odds of teeth cleaning/brushing were higher among participants living in the rural plains region (*terai*) bordering India, compared to those in the mid-hill regions, and higher among those with higher educational backgrounds [11]. Additionally, while women were more likely than men to report seeking dental care services, only 4.8% of these women had seen a dentist in the past 6 months [11].

Formative research studies from Nepal are necessary to inform the design of context-appropriate interventions and programmatic approaches. One study conducted in Newalparasi, Nepal used the Theory of Planned Behavior [12] model to better understand oral hygiene and perceptions, and decisions related to care seeking. The study found that perceived self-efficacy in relation to oral hygiene practices and expected social outcomes of having healthy teeth were important predictors for oral health behavior [13]. For example, researchers found that once-daily tooth brushing was associated with the bathing ritual, which has a symbolic meaning of creating purity [13]. There are few studies on the particular aspects of oral hygiene behavior, perceptions, and attitudes among pregnant women. This group is of particular interest given the often observed association in other settings between periodontal disease during pregnancy and perinatal outcomes [14–18]. Therefore, in Sarlahi District, Nepal, we aimed to characterize these issues through in-depth interviews (IDIs) and focus group discussions (FGDs) among pregnant and recently-delivered women. Specifically, we aimed to understand dental care-seeking patterns, practices of oral hygiene, attitudes, and knowledge relevant to oral health of pregnant and recently-delivered women.

## Methods

### Data collection

This project was conducted by the Nepal Nutrition Intervention Project – Sarlahi (NNIPS), a multi-institution research collaboration that has been conducting community-based studies in rural Sarlahi district over the past 28 years. Between August and December 2016, we conducted 16 IDIs and 3 FGDs among pregnant and recently-delivered women in this setting using purposive sampling to identify participants.

For IDIs, we selected pregnant women enrolled in an ongoing randomized pilot trial of the acceptability of oral rinses (clinicaltrials.gov: NCT02788786). Four IDI participants were selected from each of three oral rinse groups, and an additional four participants were selected from a group of women randomized to no rinse. The rinse group individuals were selected to reflect diverse levels of adherence and reactions to taste, based on preliminary data available from the trial.

The pilot trial was being conducted in communities representing only one of the two broad ethnic groups in the region. To address this, our selection of women for the FGD phase of the research leveraged a population-representative database of reproductive aged women who were current (i.e. pregnant) or previous (i.e. recently-delivered) participants in a large community-based randomized trial of topical emollient therapies and neonatal health outcomes (clinicaltrials.gov: NCT01177111). These women were purposively selected to broaden representation to both major ethnic groups within the community: Madhesi women (*n* = 2 FGDs, originally from the northern plains of India and southern Nepal) and Pahadi women (*n* = 1 FGD, originating from Nepal’s hill regions). Women were excluded if they had already participated in the IDIs or the pilot trial of oral rinses.

Prospective participants were approached by field staff to assess interest in participation, and an individually signed consent was obtained prior to initiating interviews or discussion groups. All IDIs and FGDs followed similar semi-structured interview guides with primarily open-ended questions and recommended probes. The IDI guide focused on oral hygiene behaviors and dental health care seeking, whereas the FGD guide additionally included questions on the symptoms and causes of tooth decay and gum disease and perceptions of dental health. Interviewers took hand-written notes during interviews; all interviews and group discussions were audio recorded. Field researchers wrote expanded field notes within 24 to 48 h of completing the interview or discussion group. Audio recordings were transcribed verbatim from Maithili (the local language) into Nepali with the aid of expanded field notes. Completed Nepali transcripts were translated into English and reviewed for any apparent errors in translation.

Prior to initiating the field activities, the NNIPS qualitative research team, consisting of female staff members with prior formal training and experience in conducting qualitative studies of a range of public health domains, received a two-week refresher training conducted by the authors (AJL, DJE). An additional training session using illustrative examples from the first two interviews was subsequently conducted to strengthen interviewer technique by one of the authors (AJL). Interviews (avg. 43 min) took place in the informants’ homes, while FGDs (avg. 74 mins) were conducted in field offices throughout the study area.

### Data analysis

Demographic characteristics of IDI and FGD participants were available as participants were enrolled in one or both of the ongoing randomized control trials that collected quantitative data. Transcripts were analyzed using a hybrid approach combining deductive thematic analysis with inductive coding during analysis enabling themes to emerge from the data [19]. An initial deductive coding structure was created based on the anticipated themes and on the content of the IDI and FGD guides. The codebook was revised using inductive coding to reflect emerging themes from initially coded IDIs and FGDs. The list of codes was then narrowed down into categories to produce a final codebook used for coding of transcripts. After each field activity, the research team used reflective memoing and debriefing sessions to analyze data quality, scrutinize assumptions and approaches to the research, and develop the codebook. Data were compiled and coded using Atlas.ti, version 7 using memos to track the process. Data across IDIs and FGDs were analyzed to identify themes related to dental hygiene methods, community oral health knowledge, and dental care seeking behavior among pregnant women. This study closely approached thematic saturation indicated by the aforementioned repeating thematic categories and lack of new coding structures during these debriefing and memoing sessions [20].

## Results

### Characteristics of IDI and FGD participants

The average age of all IDI and FGD participants was 20 years (Table 1). Among the 16 IDI participants, all were Madhesi, were pregnant in either their second (*n* = 10) or third (*n* = 6) trimester at the time of the interview, and had 0 to 3 prior children. Almost half (*n* = 7) reported no formal education. The three FGDs included 23 participants, fourteen of whom were pregnant, whereas the remaining nine women were in various stages of the post-partum period. A majority of FGD participants had no formal education, and number of prior live births ranged evenly from 0 to 2 (where one participant had 4).

**Table 1:** Demographic Characteristics of IDI and FGD Participants

	IDIs [*n* = 16]		FGDs [*n* = 23]	
Age (years)				
- Mean	20		21	
- Median	19		21	
- Range	16–29		15–33	
Previous live births	Frequency N (%)			
- 0	5	(31)	8	(35)
- 1	5	(31)	7	(30)
- 2	3	(19)	7	(30)
- 3	3	(19)	–	–
- 4	–	–	1	(4)
Gestational Age				
- 0-3 months	–	–	–	–
- 4-6 months	10	(63)	10	(43)
- 7-9 months	6	(38)	4	(17)
Women’s Education				
- No Schooling	7	(44)	12	(52)
- Years 1–9	8	(50)	9	(39)
- 10-SLC Pass	1	(6)	3	(13)
Ethnicity				
- Madhesi	16	(100)	16	(70)
- Pahadi	–	–	7	(30)

### Oral hygiene

Madhesi participants (i.e. all IDI participants and those from the Madhesi-comprised FGDs) reported that their usual teeth cleaning practice was to use either a toothbrush and toothpaste or *datuwan*, a teeth cleaning twig that serves as both a toothbrush and toothpaste, once a day prior to their morning meal. In contrast, women in the Pahadi FGD stated that the norm in their communities was for women to brush their teeth twice a day if not more.

IDI participants reported using either a toothbrush with Colgate (a toothpaste brand commonly found throughout shops and markets in the area) or “Dabur Lal Danta Manjan”, an ayurvedic toothpaste without fluoride, or using *datuwan*. IDI participants used the word “medicine” to describe the toothpaste used for their tooth cleaning routine, which upon further probing was revealed to be ‘Colgate’ characterized by the red color of the brand. Within the Madhesi community, women used any available *datuwan* when doing farm work, but the other types of *datuwan* used included bamboo, mango, neem, snake’s tail or chaff flower and black honey shrub (*sikat*) for specific purposes. For example, bamboo *datuwan* was reported by the participants to help whiten teeth, whereas women stated that the bitter taste of the black honey shrub helped to kill germs within the teeth. Mango *datuwan* was deemed “holy” as it can be used to clean teeth during fasting ceremonies since it is a twig of a fruit tree. This contrasts with the use of toothbrushes, which might have been in contact with meat and fish during prior brushings, contact that would be perceived as breaking one’s fast***.*** Other dentifrices mentioned included sand, mud/dirt, wood ash, and charcoal. Among the Pahadi community, women said that they used charcoal to whiten their teeth, and the types of *datuwan* used differed from the Madhesi community and included kamala tree (*sindure*), Bombay rosewood (*sisoo*), *Jatropha curcas* (*bagandi*), and Sal tree (*sakhuwa*).

When asked regarding motivations for cleaning one’s teeth twice per day, Madhesi and Pahadi community members had similar reasons including reducing the risk of caries, overall cleanliness, and preventing infections. By keeping the mouth clean, one also prevented the development of other diseases:

*It is possible to prevent some diseases by brushing your teeth. If you don’t brush your teeth, many different types of germs enter the mouth and spoil the teeth. It goes inside the stomach and [causes] diseases. It is because of the teeth that many diseases occur.*

**--6 months pregnant, Madhesi FGD participant.**

Women predominately learned about and developed their teeth cleaning routines from their parents, but others reported learning by watching people in their community, through radio and television advertisements, or indicated they were self-taught. In terms of maintaining more frequent brushing (i.e. twice per day), IDI participants identified the most pertinent barrier to be accessibility to a toothbrush and toothpaste. Many indicated, however, a willingness to increase frequency if instructed by community-based workers, such as NNIPS staff. In both Madhesi and Pahadi communities, other commonly mentioned barriers were lack of time due to work obligations, laziness, sleepiness, forgetfulness, and lack of awareness. In addition, Madhesi women said that some community members justified brushing only once daily because a toothbrush could “ruin their gums”; it was also suggested that the rough feel of a toothbrush on teeth or swollen gums could lead to women preferring to use *datuwan*.

### Symptoms, causes, and severity of tooth decay and gum disease

Women generally felt confident describing the symptoms and causes of tooth decay, and toothache and swelling in gums were commonly listed as key symptoms of tooth decay. Madhesi women additionally described a sensation of *saksakauncha (*“itching”) within the teeth that was accompanied by tingling, “swelling in teeth”, lack of appetite, and feelings of nausea or vomiting. Pahadi women additionally listed black patches on teeth, holes in teeth, bad smell, and sensitivity when drinking water as signs of tooth decay. Not rinsing the mouth after eating sweets or getting food stuck between teeth were reported as potential causes of tooth decay; some women in the Pahadi focus group also reported that decay had a familial basis, where mothers could pass the condition to their children. Madhesi women suggested other possible causes included leftover food causing “a wound in the gums or tooth creating a ground for germs to infect other teeth”, not brushing teeth before eating, eating eggplant or fish while having a toothache, chewing tobacco, drinking alcohol, or misusing a “toothpick to create a hole in a tooth”.

When discussing the symptoms and causes of gum disease, however, women were not as forthcoming as they were when talking about tooth decay. Swollen gums and pain in the gums were listed as symptoms. While Madhesi women added “swelling of the teeth”, specifically in the wisdom teeth area, and toothache as signs of gum disease, Pahadi women described a throbbing pain due to pus collecting within the gums. Madhesi women suggested that gum disease could result from wounds inflicted by poking the gums with *datuwan,* swollen gums due to caries, not brush teething before eating, allowing impurities within the mouth to cause disease, or drinking sweet tea after brushing. When similarly queried regarding cause of gum disease, Pahadi women described not knowing about gums, and could not provide any specific causes.

Women in the Madhesi community reported that children, the elderly, tobacco users, and poor people were more likely to suffer from tooth decay or gum disease. Some of these women felt strongly that poor people were at a disadvantage because they lacked the resources to buy a toothbrush and thus were forced to use *datuwan.* When prompted about the importance of oral health relative to other types of health issues, Madhesi women deemed that tooth decay and gum disease were very serious health problems because toothaches are extremely painful, can cause other diseases to manifest, and if toothache is a continuous problem, one may need to have their teeth removed, thus losing their ability to eat. In contrast, women in the Pahadi focus group expressed doubt as to the seriousness of tooth decay and gum disease, given the ubiquitous nature of the issue within their community; these women did acknowledge that such opinions, however, differed among individual community members.

### Dental healthcare seeking

When experiencing tooth and/or gum pain, participants frequently indicated that they would seek “English” or “modern” medicine either from a local shop or from an allopathic provider if brushing one’s teeth with toothpaste did not resolve the problem. Ibuprofen and paracetamol were mentioned as treatments to reduce or remove tooth pain. Several participants in both IDIs and FGDs indicated they would also consider going to a clinic or hospital. Other participants described that to alleviate tooth pain they would first get *jhar fuk,* a traditional approach where a healer performs a spell to chase “worms” (germs) out of the teeth. If this effort was unsuccessful, they would then seek allopathic medicine and/or medical treatment from an allopathic doctor. Another common treatment option for tooth pain was application of clove oil or “*sancho*,” a blend of Himalayan essential oils, which is a multipurpose herbal medicine used for the common cold, cough, body aches, and other illnesses. Pahadi women mentioned that people get fillings out of cement or silver when they have tooth pain, and if the filling is of poor quality, people are forced to get their teeth extracted.

Upon further probing as to where one could find a dentist, most IDI participants could not say with certainty where to go because they had never experienced pain that would warrant such care-seeking. Women said they would seek the counsel of their neighbors and community members on where to purchase medicine for tooth and/or gum pain, where to find a good doctor, and where to find a dentist. Among all IDI and FGD participants, no women reported ever visiting a dentist; one FDG participant summarized as follows:

*Some may have said [they didn’t need a dentist] because of lack of money. Some might have ignored it [the pain]. Some might have taken care of it and might have become all right after using herbal medicine. Some might have extracted the tooth with the cavity and thrown it away after breaking it. Some may not have been able to go because of other compulsions/ problems. Some guardians do not take them. They might have brought some tablets and said that this would make it all right. Some might have been told about herbal medicine and they must have taken it and become all right.*

**-- 6 months pregnant, Madhesi FGD participant.**

Even though participants expressed interest in seeing a dentist or doctor regarding tooth and/or gum pain, only a minority said that they would seek care in the absence of pain. Many stated that it did not make sense for them to treat a problem such as black spots on their teeth or bleeding gums if there was no pain. Instead of seeking medical attention, most women said they would either brush their teeth with medicine (toothpaste) or take no action and wait for the problem to self-resolve. Madhesi FGD participants described that when gums are very red, swollen, and/or bleeding without pain, people rinsed their mouths with hot water, applied mud or clove oil, purchased oral medicine, and went to the hospital for treatment of teeth. Pahadi women indicated that some people attempted to reduce bleeding through brushing with cooking oil and/or salt, or using *datuwan* made of *J. curcas*.

### Pregnancy and oral hygiene

Many IDI participants, as well as FGD participants from both as both ethnic groups, did not report changes in teeth cleaning routines during pregnancy. All women agreed that keeping the mouth clean was essential in preventing diseases, but there were mixed views on the association between good oral hygiene during pregnancy and healthy birth outcomes. In one of the Madhesi FGDs (but not the other FGDs), women stated that poor oral hygiene causes cavities that enter the abdomen and negatively affect the baby, and that toothaches are a serious health problem because one does not eat, which can cause the child to become “lean and thin.”

## Discussion

Findings from this qualitative study highlight important implications regarding pregnant women and oral hygiene within rural Nepal. First, while tooth brushing is commonly practiced, the normal practice was limited to once daily, and consistent use of toothbrushes and toothpaste was lacking. Knowledge of symptoms and causes of tooth decay and gum disease were limited and varied substantially across community members. This suggests that standardized, culturally-specific, and simple educational messaging need to be developed and delivered through appropriate behavioral change communication approaches. One such possibility includes training rural women in oral health promotion activities, an approach that has demonstrated improved oral health knowledge among female community members in Nepal [21].

Second, formal preventative maintenance remains a largely unknown concept in this sub-population, and dental care health seeking is almost entirely driven by a direct response to tooth and/or gum pain. In the absence of pain, a majority of women stated it was unnecessary to seek a dentist because teeth brushing would be sufficient to address bleeding gums or spots on their teeth in cases where the problem would not resolve itself. Delay in care-seeking among this population is not unique to oral health and pregnant women. For example, among the same community in Sarlahi district, delays in care-seeking for maternal and newborn complications have been attributed to low perceived severity of the illness even when symptoms were recognized early [22]. In these cases, care was initially sought from informal health providers including traditional birth attendants, traditional healers, and village doctors whereas barriers to seeking care from any health facility included transportation, finances, and distance to the facility [22]. These care-seeking findings for maternal and newborn complications suggest that it is norm for pregnant or recently delivered women in the Sarlahi district to delay care-seeking from formal providers and/or health facilities for many health problems including ones related to oral health. Moreover, care-seeking for maternal illness improves significantly in low and middle income countries when antenatal or postnatal counseling stresses illness recognition and referrals by community health workers [23]. By changing the way illness is perceived in these low income settings and building a solid referral foundation using community health workers, the norm of delaying care-seeking among pregnant and recently delivered women can be changed.

Third, limited access to qualified providers tends to delay treatment seeking until pain is severe and/or home or traditional remedies have failed. When more formal care is sought, women often expressed going to a “hospital for treatment of teeth,” of which there were two in this area at the time of data collection. While one of these was staffed by a practitioner with a dental certificate, neither practitioner was a fully trained or qualified dentist. During the study period, the nearest qualified dentists were in the neighboring districts of Janakpur and Birgunj, located 80 km and 100 km to the east and west, respectively. Public transportation to and from one of these providers would require a full day (or more if the providers were not able to immediately see clients). Given these substantive distance barriers, women (and the broader community) would most benefit from the improvement of local facilities through formal dental training of local practitioners and from increasing the number of qualified dentists in the area.

A limitation of this study is that our interviewers found it challenging to effectively probe women in this community about oral health, in order to elicit richer responses. These challenges in engaging respondents in conversation may not be surprising given that young women in this community, especially among the Madhesi, are frequently reticent to share their opinion or be assertive. Interviewers frequently commented that IDI participants were reluctant to speak about their oral health for fear of saying something incorrect (social desirability bias) or because they claimed that they had no knowledge on the subject. This challenge may have resulted in interviewers resorting to more direct or leading questions. Interviewers were women of the community an in approximately similar age range as participants, but there other characteristics about the interviewers (for example, cultural deference towards “guests”; formal employment in non-agricultural or “skilled” work can confer an extra level of respect, etc) that might have inhibited more full sharing of information or opinions.. Facilitators of FGDs indicated that the group setting allowed women to feel more comfortable not only expressing their opinion, but contrasting their viewpoints with those of others in the group. In addition, as some IDI respondents had also participated in the ongoing oral rinse pilot trial, exposure to positive oral health behavior messages might have influenced their responses; it is possible that other IDI or FGD participants who had not participated in that trial might also have indirectly been exposed to similar content. The impact, however of such exposures is likely limited, as interviewers were instructed to parse out behavior changes related to involvement in the oral health trial through effective probing during each interview, and analysis of transcripts did not indicate substantively different knowledge or attitudes regarding, for example, oral hygiene practices between those who did or did not participate in the rinse trial In terms of generalizability, while the knowledge, attitudes, and practices related to oral hygiene and care-seeking are likely generalizable to broad population in this region, this specific population might be more amenable to behavior change communication approach, given that NNIPS has been working closely with these communities for 28 years. Specifically their future readiness to engage in and follow instructions related to improved oral health behaviors such as improved frequency of brushing may be overestimated relative to other populations.

## Conclusions

We found that pregnant or recently-delivered women in the Sarlahi community either brushed their teeth with a toothbrush and toothpaste once daily or used a teeth cleaning twig, but were receptive to switching their routine to brushing twice daily with toothpaste when instructed by a health worker. Women in this community were unable to correctly identify the signs and causes of tooth decay and gum disease and lacked knowledge on where to find qualified dentists within the study area. Based on our findings, we suggest that future efforts involving oral health and pregnant women in low-income settings focus on providing tools and resources to maintain dental hygiene, promoting good oral health behaviors and knowledge (possibly through context-appropriate cadres of trained community-based workers), and increasing access to qualified and fully trained dentists to improve the overall oral health of pregnant women.

## Abbreviations

FGD: Focus group discussion
IDI: In-depth interview
NNIPS: Nepali Nutrition Intervention Project Sarlahi
